# Alternatively spliced *BobCAL* transcripts alter curd morphotypes in a collection of Chinese cauliflower accessions

**DOI:** 10.1038/s41438-020-00378-x

**Published:** 2020-10-01

**Authors:** Wenguang Cao, Biting Cao, Xuan Wang, Jinjuan Bai, Yong-Zhen Xu, Jianjun Zhao, Xiaorong Li, Yuke He, Shengwu Hu

**Affiliations:** 1 National Laboratory of Plant Molecular Genetics, Center for Excellence in Molecular Plant Sciences, Shanghai Institutes of Plant Physiology & Ecology, Chinese Academy of Science, 300 Fenglin Road, Shanghai, 200032 China; 2 State Key Laboratory of Crop Stress Biology in Arid Areas and College of Agronomy, Northwest A&F University, Yangling, Shaanxi China; 3 State Key Laboratory of North China Crop Improvement and Regulation, Department of Horticulture, Hebei Agricultural University, Baoding, 071001 China

**Keywords:** Plant morphogenesis, Non-model organisms

## Abstract

The curd of cauliflower (*Brassica oleracea* L. var. *botrytis*) is a modified inflorescence that is consumed as a vegetable. Curd formation is proposed to be due to a mutation in the *BobCAULIFLOWER* (*BobCAL*) gene, but the genetic relationship between *BobCAL* variation and curd morphotypes remains obscure. To address this question, we collected and classified a collection of 78 cauliflower accessions into four subpopulations according to curd surface features: smooth, coarse, granular, and hairy curd morphotypes. Through the cDNA sequencing of *BobCAL* alleles, we showed that smooth and coarse accessions characterized by inflorescence meristem arrest presented a strong association with the 451T SNP (*BobCAL_T*), whereas granular and hairy accessions marked with floral organ arrest presented an association with 451G (*BobCAL_G*). Interestingly, all *BobCAL* alleles were alternatively spliced, resulting in a total of four alternative splice (AS) variants due to the retention of the fourth and/or seventh introns. Among accessions with *BobCAL_G* alleles, the total expression of all these AS variants in granular plants was almost equal to that in hairy plants; however, the expression of the individual AS variants encoding intact proteins relative to those encoding truncated proteins differed. Hairy accessions showed relatively high expression of the individual variants encoding intact proteins, whereas granular accessions displayed relatively low expression. In smooth cauliflower, the overexpression of the *BobCAL_Ga* variant caused an alteration in the curd morphotype from smooth to hairy, concurrent with an increase in the expression levels of downstream floral identity genes. These results reveal that alternative splicing of *BobCAL* transcripts is involved in the determination of cauliflower curd morphotypes.

## Abstract (web summary)

Four types of curd morphotypes were characterized from a collection of 78 cauliflower accessions, from which 4 splice variants of *BobCAL* alleles were identified, and their relative expression levels were defined to determine curd morphotypes.

## Introduction

The species *Brassica oleracea* includes several cultivated subspecies with extreme morphological divergence of various organs due to the proliferation of different types of meristems. Among these subspecies, cabbage (*B. oleracea* L. var. *capitata*), kale (*B. oleracea* L. var. *acephala*), and Brussels sprouts (*B. oleracea* L. var. *gemmifera*) exhibit elongated inflorescences, while cauliflower (*B. oleracea* L. var. *botrytis*) and broccoli (*B. oleracea* L. var. *italica*) are characterized by inflorescences that are modified into large dense structures (curds). These varying forms are due to selection for different characteristics during domestication^1^. Cauliflower curd is one of the most important vegetable products. Curd induction and development have been previously investigated by a number of researchers (for reviews^1,2^). Although curd formation was originally considered to be a vegetative process^3–6^, the typical curd is now considered to be a dense mass of stage-arrested inflorescence/floral meristems derived from their own iterative proliferation^7,8^. At the curd formation stage, the extension of the inner inflorescence branches is restricted, while the inflorescence/floral meristems on the surface temporarily lose the ability to generate floral organs and instead repeatedly copy themselves. This arrest of the floral organ generation ability persists until external conditions, such as temperature and day length, and internal cues are appropriate for continued floral development. The exploration of the mechanisms controlling the cauliflower curd phenotype can contribute to the improvement of product quality and aid in the understanding of the general control of floral development. In regard to this goal, little progress had been achieved until the *CAULIFLOWER* phenotype, resembling the cauliflower curd, was identified and characterized in the *ap1 cal* mutant of *Arabidopsis thaliana*, a close relative of cauliflower^9,10^.

In the *ap1 cal* double mutant, floral meristems that give rise to flowers in the wild type instead behave as inflorescence meristems, which in turn produce higher-order inflorescence meristems in a phyllotactic spiral on their flanks^9,11^. As a result, these plants fail to undergo the normal inflorescence-to-floral transition and only occasionally produce floral organs.

*CAL* and *AP1* are closely related genes that share redundant roles in the specification of floral meristem identity^12^. Both genes encode MADS-domain proteins composed of four different domains, designated as MADS (M), intervening (I), keratin-like (K) and C-terminal (C). Studies of MADS-domain proteins in diverse species have shown that AP1 and CAL can each interact with a shared set of proteins, including SEPALLATA3 (SEP3), SOC1, SVP, and AGL24^13–16^. In addition, the M domain is responsible for binding to DNA. The I region participates in homodimer formation^17,18^. The K domain has been implicated in protein–protein interactions^17–21^ and is postulated to form several amphipathic α-helices, referred to as K1, K2, and K3. K2 and K1 are required for many interactions, such as PI/SEP3 (or PI/SEP1) interaction^22^. The C-terminal region has been proposed to be involved in transcriptional activation^23^.

Consistent with the observed similarities between *A. thaliana* and cauliflower curd phenotypes, a mutation has been found in *BobCAL*, a *CAL* gene ortholog in cauliflower considered to be associated with curd formation^10^. In a study of *B. oleracea*, a nonsense mutation in exon 5 of the *BoCAL* gene was shown to be nearly fixed in most accessions of cauliflower and broccoli but was also present in some accessions of cabbage and kale, which do not produce curds. The mutant allele has also been found in some broccoli and noncurding *Brassica* accessions^24,25^. On the other hand, the wild-type *BobCAL* allele without this nonsense mutation can occur in cauliflower^24^. A subsequent survey of broccoli and cauliflower accessions found only a weak association between *BobCAL* mutant alleles and cauliflower phenotypes^26^. These results suggest that the molecular mechanism underlying curd phenotypes is more complicated than expected.

To understand the genetic relationship between *BobCAL* and curd phenotypes, we classified a collection of cauliflower accessions and cloned the cDNAs of their *BobCAL* genes. We first distinguished granular and hairy curd phenotypes from this population according to curd texture and the curd surface. cDNA sequencing then revealed that some accessions with granular or hairy curd phenotypes lacked the *BobCAL* nonsense mutation. To our surprise, several alternative splice (AS) variants were identified from the different *BobCAL* alleles. More importantly, we found that differences in expression levels among these splice variants were the molecular cause of the granular and hairy curd phenotypes. This conclusion was verified by the overexpression of a *BobCAL* variant with normal function. We have thus determined that the AS of *BobCAL* alleles alters curd phenotypes.

## Results

### Variation in curd morphotypes

**Fig. 1:**
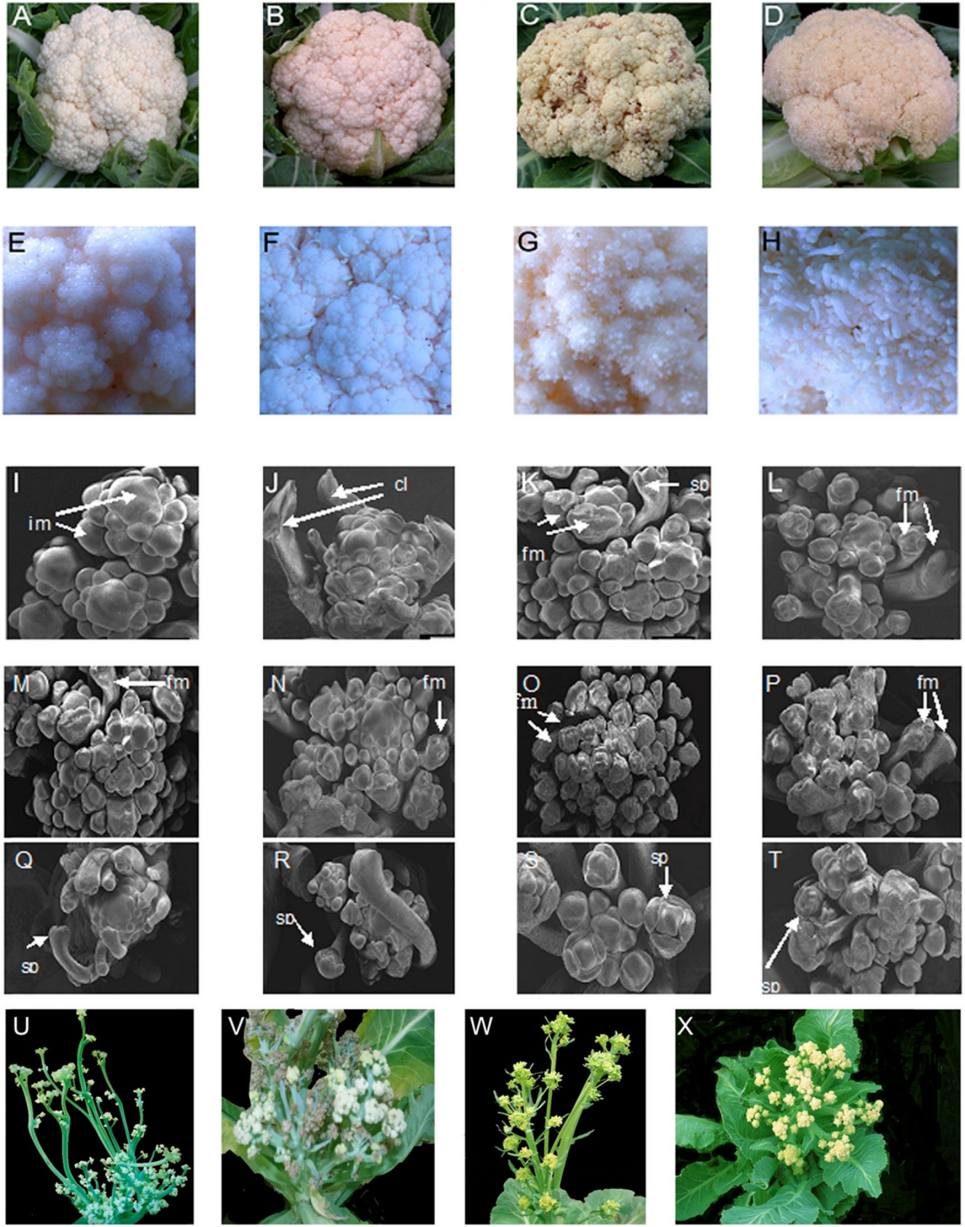
Curds of four types of cauliflower accessions Morphotypes of smooth (**a**), coarse (**b**), granular (**c**), and hairy (**d**) curds of cauliflower at the curd maturation stage. Surfaces of smooth (**e**), coarse (**f**), granular (**g**), and hairy (**h**) curds. **i**–**t** Scanning electron micrographs of inflorescence, floral, and organ meristems. Inflorescence meristems of smooth (**i**) and coarse (**j**) curds and floral meristems of granular (**k**) and hairy (**l**) curds at the curd enlargement stage. Inflorescence meristems of smooth (**m**) and coarse (**n**) accessions and floral meristems of granular (**o**) and hairy (**p**) accessions at the bolting stage in curds during winter. Flower buds of smooth (**q**) and coarse (**r**) accessions and floral meristems of granular (**s**) and hairy (**t**) accessions at the flowering stage, immediately after bolting. Inflorescences of smooth (**u**) and coarse (**v**) accessions and floral meristems of granular (**w**) and hairy (**x**) accessions at the bolting stage in curds during winter. Arrows indicate meristems or floral organs. im inflorescence meristems, cl cauline leaves, sp sepal primordia, fm floral meristems.

In this study, 78 cauliflower accessions were collected from different sources. To avoid heterogeneity, all accessions were self-fertilized for more than five generations, and each accession was represented by an inbred line. These inbred lines were grown in the field during the same seasons in 2016–2018. We measured curd-related traits such as developmental arrest, the width of curds, the number of days to curd maturity, and the number of days to flowering. The parameters of curd-related traits did not exhibit distinct differences between the years. Hence, we used the parameters recorded in all 3 years for the analysis of frequency distribution (Supplementary Fig. 1). The variance in the curd-related traits between the accessions was high. To analyze the genetic basis of their curd phenotypes, these accessions were classified according to their curd texture into two categories: tightly compact or loosely compact (Fig. 1a–d). Tightly compact curds were difficult to divide by hand, whereas those classified as loosely compact came apart easily. On the basis of curd surface features, accessions with tightly compact curds were subclassified into smooth curd (*sc*) and coarse curd (*cc*) subpopulations (Fig. 1e–h), while those exhibiting loosely compact curds were divided into granular curd (*gc*) and hairy curd (*hc*) categories. The number of accessions in each subpopulation was 41 in *sc*, 16 in *cc*, 10 in *gc* and 11 in *hc* (Table 1), where *sc* corresponds to the “classic” curd phenotype, accounting for 50% of the total accession.

**Table 1:** Association of curd phenotypes with the G451T single-nucleotide polymorphism in a collection of 78 cauliflower accessions.

Curd phenotypes	Number of accessions with		
	*BobCAL_T*	*BobCAL_G*	Sum
Smooth	38	3	41
Coarse	15	1	16
Granular	0	10	10
Hairy	1	10	11
Total	54	24	78

We next examined the progress of curd development. At the early reproductive stage, the inflorescence meristems of smooth curds resembled broad domes from which secondary inflorescence meristems arose and rapidly became more prominent (Fig. 1e). Each secondary inflorescence meristem functioned as the apical meristem of a secondary branch and started to produce tertiary inflorescence meristems, which in turn became the apical meristems of tertiary branches. Under the microscope, numerous arrested secondary inflorescence meristems were observed regularly arranged around the main inflorescences, while the inflorescence meristems remained terminal (Fig. 1i). In addition, distinct stratified layers were easily recognized in the inflorescence meristems. These observations indicate that the inflorescence meristems of smooth curds were arrested and did not produce floral meristems. The first- and higher-order inflorescence meristems of coarse curds looked similar to those of smooth curds (Fig. 1f). They differed in that the cauline leaves grew vertically and protruded from the curd surfaces, resulting in coarse curd surfaces where naked floral meristems were visible (Fig. 1j). In contrast, on the surfaces of granular curds, inflorescences of different orders developed further and produced floral meristems (Fig. 1g). Although sepal, petal, stamen, and pistil primordia were initiated, they failed to develop into floral organs, and this interesting phenomenon suggested that floral meristems were arrested. Under the microscope, numerous arrested floral meristems were observed around the main inflorescences, with some cauline leaves protruding between branch inflorescences and arising from the curd surface like thorns (Fig. 1k). Many floral meristems appeared on the surface of hairy curds (Fig. 1h). The floral organs were initiated and developed into tiny floral buds; however, these floral buds stopped growing and resembled hairs, indicating that the floral organs were arrested. Under the microscope, arrested floral organs and aberrant floral buds were clearly visible (Fig. 1l).

The cauliflower development process can be broadly divided into seedling, curd formation, and flowering stages. In the field, curds that were fully mature before winter did not increase further in size. The following spring, a few inflorescence meristems began to develop and gave rise to new floral meristems on their periphery. The main and secondary inflorescence meristems in smooth curds elongated without the formation of cauline leaves (Fig. 1m, q), concurrent with the differentiation of a few floral buds. In coarse curds, the elongation of inflorescence meristems subtending cauline leaves and the differentiation of a few floral buds took place along with the growth of cauline leaves (Fig. 1n, r). Granular curds resumed development following floral meristem arrest and generated numerous floral buds that later flowered (Fig. 1o, s). When the floral meristems and internodes of inflorescences within curds elongated, the curds became “loose”, and a few floral meristems developed into floral primordia, signaling the curd bolting stage. Floral meristems developed slowly but continuously, and sepals, petals, carpals, and stamens then began to form in preparation for normal flowering. At the same time, the curd surface became green because of the appearance of green sepals. Hairy curds resumed development following floral organ arrest and quickly flowered (Fig. 1p, t).

Plants of different accessions were transplanted into a growth chamber (16/8-h photoperiod with irradiance of 200 μmol photons/m^2^/s and a growth temperature of 25 °C) for further development. Under these conditions, which were conducive to flowering, the arrest of inflorescence meristems, floral meristems and floral organs was gradually broken. In *sc* and *cc* accessions, floral stems elongated, and a few floral buds developed, while most of the original inflorescence meristems remained suppressed or withered at the stage of primary protuberance (Fig. 1u, v). In contrast, inflorescence elongation in *gc* and *hc* accessions was concurrent with the development of floral organs (Fig. 1w, x).

### Variation in *BobCAL* alleles

To detect the mutations of *BobCAL* alleles in cauliflower accessions, we performed an extensive PCR analysis to amplify *BobCAL* genes in the genomes of *sc*, *cc*, *gc*, and *hc* accessions. Some accessions were found to be heterozygous. To obtain homologous genotypes to achieve the highest discriminatory accuracy, all accessions were self-fertilized for more than five generations, and each accession was represented by one inbred line.

To examine the variations in *BobCAL* alleles, we cloned their coding regions using a primer pair (BobCAL_F/BobCAL_R; Supplementary Table 1) designed to span the complete coding sequence. We identified 266 variant sites in the coding sequences of *BobCAL* alleles and 19 single-nucleotide polymorphisms (SNPs) across the collection of 78 accessions (Supplementary Table 2). The most interesting SNP was SNP451, which varied between G and T: while *BobCAL* containing 451G (referred to as *BobCAL*_*G*) encoded a full-length protein, that containing 451T (*BobCAL_T*) encoded a truncated version because of a premature stop codon introduced by the SNP.

Among 57 accessions with tightly compact curds, 92% (38/41) of *sc* accessions and 94% (15/16) of *cc* accessions were found to carry 451T (Table 1). In contrast, all (10) *gc* accessions and 91% (10/11) of *hc* accessions harbored 451G. These results suggest that the 451T SNP is highly associated with tightly compact curds, while 451G is correlated with loosely compact curds. To our surprise, a few *sc* and *cc* accessions contained 451G, and one *hc* accession possessed 451T, revealing that 451G is not the only genetic factor responsible for the loosely compact curd phenotype.

### Alternative splicing of BobCAL genes in cauliflower

The *BobCAL* gene contains 8 exons encoding 251 amino acids. *BobCAL* alleles in different cauliflower accessions exhibited very similar structures and gene sequences. The *BobCAL_G* gene was found to encode a full-length protein, whereas *BobCAL_T* harbored a premature termination codon in exon 5 due to the 451T SNP. To determine the relationship between the gene sequences and curd morphotypes of different accessions, we performed extensive cloning and sequencing of *BobCAL* cDNAs.

**Fig. 2:**
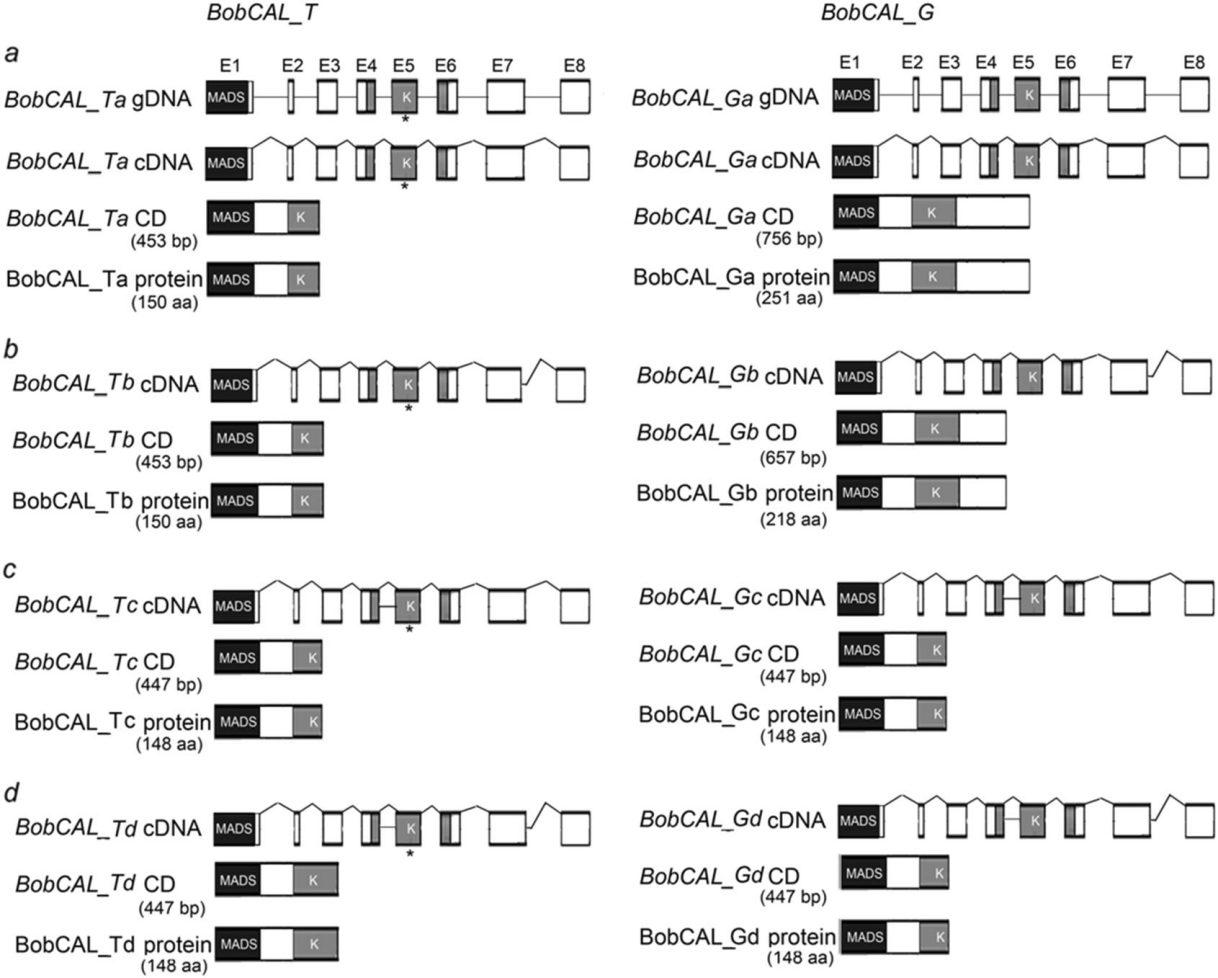
Diagrams showing the alternative splicing of *BobCAL_T* and *BobCAL_G* alleles. The AS variants of *BobCAL_T* and *BobCAL_G* are indicated by the suffixes *a*, *b*, *c*, and *d*. Gene structures, splice variants, coding sequences and putative proteins are shown. Exons are symbolized by boxes and introns by horizontal lines. Boxes show exons and domains, while straight lines indicate introns. Asterisks indicate the 451T SNP. MADS- and K-boxes appear in black and gray, respectively.

The coding sequences of *BobCAL_G* alleles in accessions with loosely compact curds were found to contain two AS sites (GenBank: AY514052–AY514055) (Fig. 2). One type of AS involved the retention of intron 4, which introduced a premature termination codon after amino acid 148. The other AS variant was an AS donor of intron 7, located 6 bp downstream from the regular 3′ splice site of intron 7, which would introduce another premature termination mutation at position 219. The AS of *BobCAL_G* was found to occur at introns 4 and 7, either individually or in combination, to yield four splice variants: *BobCAL_Ga*, *_Gb*,*_Gc* and *_Gd*. *BobCAL_Ga* (variant *a* of *BobCAL_G*), comprising 756 bp, was predicted to encode a full-length protein of 251 amino acids, while the 762-bp *BobCAL_Gb* (variant *b*) encoded a 218-amino-acid putative protein; the 447-bp *BobCAL_Gc* (variant *c*) retained intron 4 and encoded a putative protein of 148 amino acids; and *BobCAL_Gd* (variant *d*), consisting of 447 bp, encoded a putative protein of 148 amino acids as well. Similarly, the four types of *BobCAL_T* AS variants perfectly corresponded to those of *BobCAL_G*, except that *BobCAL_Tb* encoded a putative protein consisting of 150 amino acids, which was 2 amino acids shorter than that encoded by *BobCAL_Ta*.

To examine the types of the AS of *BobCAL* alleles, we performed RT-PCR using strand-specific primers designed to amplify AS variants (Supplementary Table 1). We detected three or four types of AS variants in each accession, with most accessions exhibiting four (Table 2). Given that *BobCAL_Ga* and *BobCAL_Gb* were functional while *BobCAL_Gc* and *BobCAL_Gd* were not, we wondered whether *BobCAL_Ga* and *BobCAL_Gb* were related to the granular and hairy curd morphotypes. All 20 *gc* and *hc* accessions possessed the *BobCAL_Ga* and *BobCAL_Gb* alleles; however, three or four types of AS variants were evident in *gc* accessions, which was also found in *hc* accessions (Supplementary Table 2). Different AS variants could thus be ruled out as the molecular factor responsible for the granular and hairy curd morphotypes.

**Table 2:** Number of different alternative splice variants detected in 78 cauliflower accessions.

Curd phenotypes	*a, b, c, d*	*a, b, d*	*a, b, c*	*b, c, d*	*a, c, d*	Total
Smooth	33	3	2	1	2	41
Coarse	14	1	0	0	1	16
Granular	8	0	1	1	0	10
Hairy	9	0	0	1	1	11
Sum	64	4	3	3	4	78

### Expression levels of splice variants of BobCAL genes

**Fig. 3:**
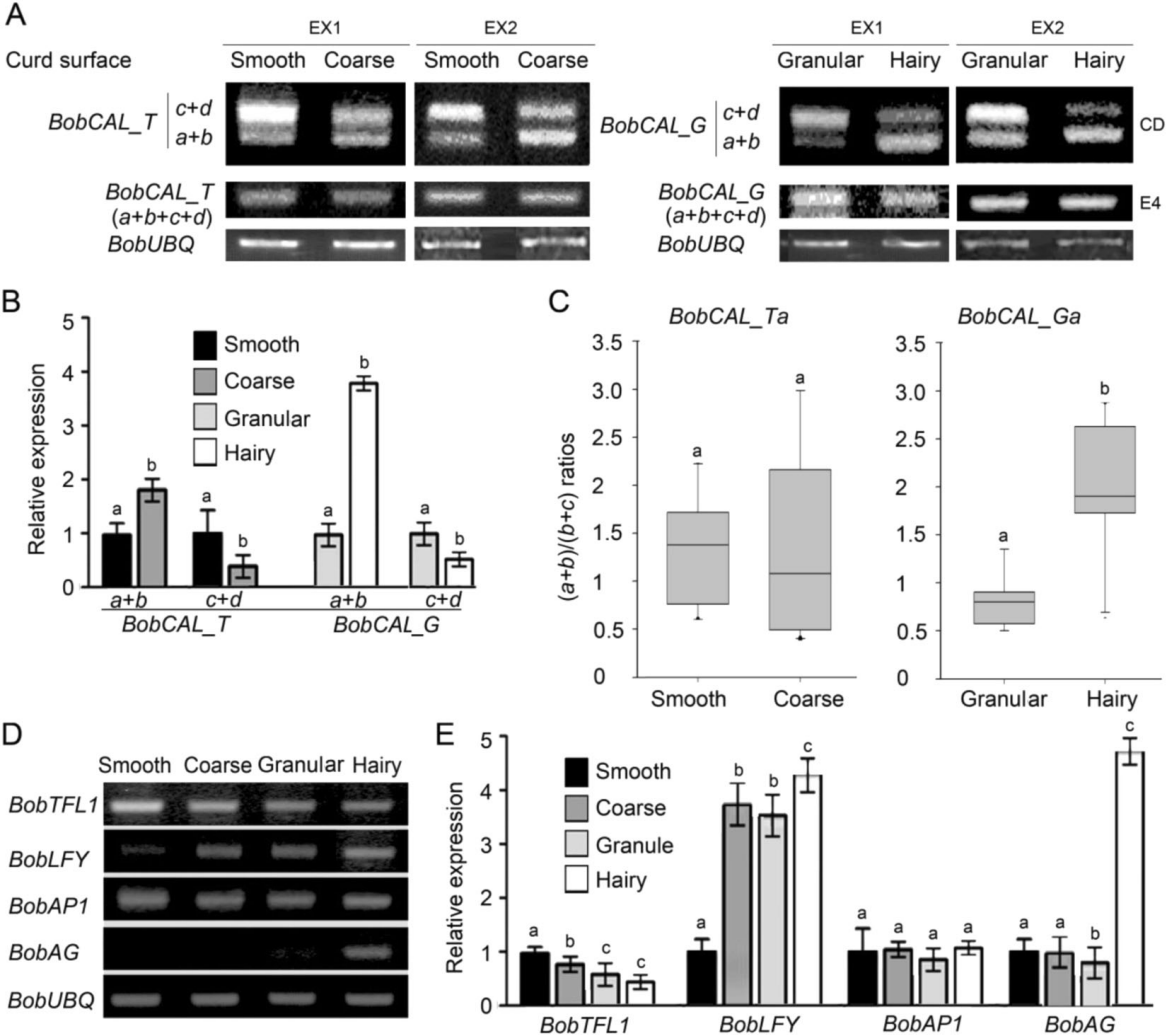
Expression of BobCAL splice variants and BobCAL-related genes in cauliflower accessions. **a** Gels showing the four types of *BobCAL* splice variants in smooth (*sc-2*), coarse (*cc-1*), granular (*gc-1*) and hairy (*hc-1*) accessions. The (*a* + *b*) band includes variants *a* and *b*, while the (*c* + *d*) band includes variants *c* and *d*. EX1, experiment 1; EX2, experiment 2; CD, coding sequence; E4, the fourth exon. **b** Expression levels of variant pairs (*a* + *b*) and (*c* + *d*). Three biological replicates of quantitative PCR were performed for each gene. The relative transcript level of each gene was normalized to that *BobUBQ* (*BobUBIQUITIN*) cDNA level for quantification. **c** Boxplot analysis showing the association between the (*a* + *b*)/(*c* + *d*) ratios of *BobCAL_T* and *BobCAL_G*. All data are presented as the mean ± SEM (*n* = 10 accessions). **d** Gels showing the expression levels of *BobCAL*-related genes in four types of cauliflower accessions. *BobUBQ* was used as an internal control. **e** Real-time PCR showing the relative expression of *BobCAL*-related genes in four types of cauliflower accessions. Three biological replicates of quantitative PCR were performed for each gene. The relative transcript level of each gene was normalized to the *BobUBQ* cDNA for quantification. Error bars indicate the standard error. Clusters with the same letters are not significantly differentiated from each other at the 0.01 level according to the *t*-test.

The *CAL* gene is strongly expressed in the inflorescence meristems of *Arabidopsis*^27^. To further clarify the relationship between the AS of *BobCAL* and curd morphotypes, we collected RNA samples from the shoot tips of cauliflower plants at the curd induction stage and analyzed the expression levels of splice variants in the different accessions by RT-PCR. In *gc* and *hc* plants, RT-PCR amplification using primer pairs spanning the complete *BobCAL_G* coding sequence (Supplementary Table 3) yielded two bands (Fig. 3a). For the expression analysis of each splice variant, we used *BobUBQ* as the internal control and performed more than three repetitions of the RT-PCR experiments to ensure that the RT-PCR methods reproducibly determined the expression levels, and the expression levels could be experimentally validated. The sequencing of these PCR products revealed that the large band corresponded to *BobCAL_Gc* and *BobCAL_Gd*, while the small band consisted of *BobCAL_Ga* and *BobCAL_Gb*. In *gc* plants, the small band was weaker than the large band, indicating that the combined number of *BobCAL_Ga* and *BobCAL_Gb* transcripts (*a* + *b*) was apparently less than that of *BobCAL_Gc* and *BobCAL_Gd* (*c* + *d*). In *hc* plants, however, the small band was stronger than the large band, indicating that the combined number of *BobCAL_Ga* and *BobCAL_Gb* transcripts (*a* + *b*) was greater than that of *BobCAL_Gc* and *BobCAL_Gd* (*c* + *d*). These results suggest that the expression levels of the *BobCAL_Ga* and *BobCAL_Gb* transcripts (*a* + *b*) relative to the *BobCAL_Gc* and *BobCAL_Gd* (*c* + *d*) transcripts in *hc* accessions are much higher than in *gc* accessions.

In all *hc* accessions except for *hc-6*, the ratios of *BobCAL_G* (*a* + *b*) to (*c* + *d*) were >1.5 (Table 3), meaning that the *hc* accessions were associated with relatively high expression of *BobCAL_Ga* and *BobCAL_Gb* transcripts (*a* + *b*). *hc-6* was exceptional in that the ratio of *BobCAL_T* (*a* + *b*) to (*c* + *d*) was >1.5. In the *gc* accessions, the ratios of (*a* + *b*) to (*c* + *d*) were ≦1.5, indicating that *gc* accessions were associated with relatively low expression of *BobCAL_Ga* and *BobCAL_Gb* transcripts (*a* + *b*). In *sc* and *cc* plants, RT-PCR also produced two bands, where the smaller band was weaker than the larger one. The larger band was composed of *BobCAL_Tc* and *BobCAL_Td*, and the short band corresponded to *BobCAL_Ta* and *BobCAL_Tb*. The combined number of *BobCAL_Tc* and *BobCAL_Td* transcripts (*c* + *d*) was apparently greater than that of *BobCAL_Ta* and *BobCAL_Tb* (*a* + *b*) transcripts. Among all *sc* accessions except for *sc-3* and *sc-14*, approximately half of the accessions presented ratios of *BobCAL_T* (*a* + *b*) to (*c* + *d*) >1.5, while the other half presented ratios ≦1.5 (Table 3); the same was true among all *cc* accessions except for *cc-10*. Accessions *sc-3*, *sc-14*, and *cc-10* harbored *BobCAL_G* alleles. These results indicated that neither *sc* nor *cc* accessions were associated with relatively high expression of *BobCAL_Ta* and *BobCAL_Tb* transcripts (*a* + *b*).

**Table 3:** Association between curd phenotypes and the ratio of the expression levels *BobCAL* variants (*a* + *b*) versus (*c* + *d*).

Splicing variants		Number of accessions				Sum
Alleles	(*a* + *b*)/(*c* + *d*)	Smooth	Coarse	Granular	Hairy	
*BobCAL_T*	>1.5	18	8	0	1	27
	≦1.5	20	7	0	0	27
*BobCAL_G*	>1.5	2	1	0	11	14
	≦1.5	0	0	10	0	10
Total		40	16	10	12	78

Real-time PCR showed that *BobCAL_Ga* and *BobCAL_Gb* (*a* + *b*) expression levels in *hc* plants were significantly higher than those of *gc* plants (Fig. 3b), while (*c* + *d*) expression levels were significantly lower than those of *gc* plants. Importantly, higher expression levels of (*a* + *b*) variants were significantly associated with the hairy curd morphotype (Fig. 3c), while lower expression levels were significantly related to granular curds. These results strongly suggest that the relative expression levels of *BobCAL_Ga* and *BobCAL_Gb* determine the granular and hairy curd morphotypes.

To investigate whether the total abundance of the different AS transcripts was the same in *gc* and *hc* plants, we performed RT-PCR using the pair of primers spanning exon 4 of the *BobCAL_G* alleles (Supplementary Table 1). The expression levels obtained using these primers were expected to reflect total abundance of the transcripts of all four AS variants in the studied plants. In the *sc* accessions, the total transcript levels of the four AS variants were roughly equal to those in the *cc* accessions (Fig. 3a). Similarly, total transcript levels were generally the same between *gc* and *cc* accessions.

To answer the question of whether the abundance of (*a* + *b*) relative to (*c* + *d*) affects curd morphotypes, we analyzed the difference between the populations of different curd morphotypes through boxplot analysis. We randomly chose 10 accessions from each population to compare (*a* + *b*)/(*c* + *d*) ratios. The difference in the (*a* + *b*)/(*c* + *d*) ratios of *BobCAL_T* between the *sc* and *cc* populations was not significant, indicating that smooth curds could not be discriminated from coarse curds according to the (*a* + *b*)/(*c* + *d*) ratios of *BobCAL_T* (Fig. 3c). In contrast, the difference in the (*a* + *b*)/(*c* + *d*) ratios of *BobCAL_G* between the *gc* and *hc* populations was significant, indicating that hairy curds could be discriminated from granular curds according to the (*a* + *b*)/(*c* + *d*) ratios of *BobCAL_G*. This result implies that *BobCAL_Ta* and *BobCAL_Tb* have lost their original function, probably because of the G451T mutation, whereas *BobCAL_ga* and *BobCAL_Gb* are functional, and the amount of *BobCAL_G* (*a* + *b*) relative to *BobCAL_G* (*c* + *d*) is responsible for the granular or hairy morphotypes.

### Expression levels of other floral identity genes

The alterative splicing of *BobCAL* may alter the expression patterns of related meristem identity genes. To evaluate the relationship between *BobCAL* and major meristem identity genes, we studied the expression patterns of the meristem genes *BobTFL1*, *BobLFY* and *BobAP1* and the floral organ identity gene *BobAG*.

At the curd induction stage, *BobTFL1* was strongly expressed in *sc* accessions but was weakly detected in *hc* plants (Fig. 3d, e). In contrast, *BobLFY* was weakly expressed in *sc* accessions and strongly expressed in *hc* accessions. In *Arabidopsis*, the inhibition of TFL1 requires CAL/AP1^27^. The strong expression of *BobTFL1* in *sc* accessions means that the truncated proteins encoded by *BobCAL_T* cannot function to inhibit BobTFL1. Conversely, the weak expression of *BobTFL1* in *hc* accessions indicates that *BobCAL_Ga* does inhibit *BobTFL* expression to some extent. In the *sc* accessions, *BobTFL1* was strongly expressed at the curd initiation stage, which indicates that early inflorescence meristems are the sites of *BobTFL1* expression in the absence of *BobCAL* activity. The expression levels of *BobLFY* in the *gc* and *hc* accessions were much higher than those in the *sc* accessions. *BobAP1* expression was almost the same in the four groups of accessions.

### Gain of function of *BobCAL_G* in smooth cauliflower

**Fig. 4:**
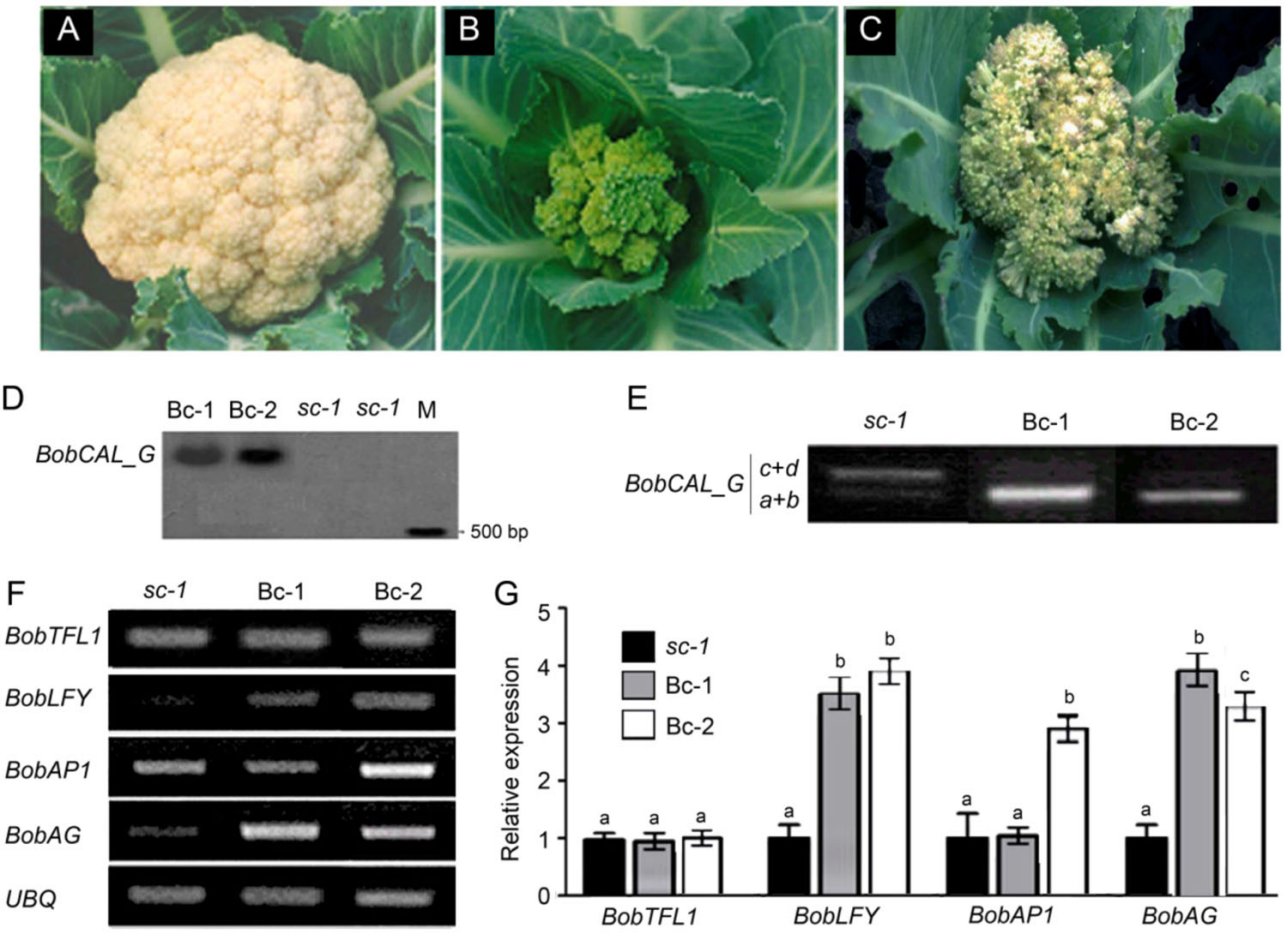
Expression of *p35S::BobCAL_Ga* in transgenic cauliflower and expression of *BobCAL* splice variants in wild-type plants **a** Wild-type plant with smooth curds. Transgenic Bc-1 plant (**b**) with hairy-like curds. **c** Transgenic Bc-2 plant with hairy-like curds. **d** Southern hybridization results for transgenic plants using a CaMV 35S promoter fragment labeled with 32P-dCTP as a probe. Lane M is a molecular size marker. Comparisons of the expression levels of *BobCAL* splice variants (**e**) and *BobCAL*-related genes (**f, g**) in transgenic plants based on real-time PCR. The relative transcript level of each gene was normalized to the *BobUBQ* cDNA level for quantification. Error bars represent the SE (standard error) calculated from three biological replicates, each of which included three technical replicates. *a* and *b* indicate significant differences at the 0.05 and 0.01 levels, respectively, according to the *t*-test.

The premature termination codon (GAG → TAG) in the fifth exon of the *BobCAL* gene has been deduced to affect the curd morphotype of cauliflower^9,10^, but direct experimental evidence of this effect has been lacking. To support this deduction, a functional *BobCAL_Ga* variant from *hc-1*, a representative inbred line of the *hc* accessions, was constructed from the T-DNA of a binary vector under the control of the CaMV 35S promotor and then introduced into *sc-1*, a representative *sc* inbred line. While the inflorescences of *sc-1* plants were severely arrested and subsequently formed smooth curds, the inflorescences of the transgenic Bc-1 and Bc-2 lines were not fully arrested and failed to yield smooth curds (Fig. 4a–c). These plants instead produced floral meristems immediately after floral initiation and underwent floral bud differentiation, which resulted in an *hc*-like curd phenotype. In both transgenic lines, green inflorescences were generated from the shoot apices of the plants, and inflorescence and floral meristem arrest was consequently not obvious. In terms of appearance, the inflorescences of the Bc-1 line exhibited a greater number of developed green floral buds than did those of the Bc-2 line; they were visually similar to cabbage inflorescences but more branched and dwarfed. Under a scanning electron microscope, we observed that the flower buds of transgenic cauliflower exhibited developing floral organs on their shoot apices, whereas the inflorescence meristems of control plants remained arrested. The most striking difference was that Bc-1 and Bc-2 plants did not require low temperatures for vernalization and began flowering in autumn, while *sc-2* plants underwent vernalization during winter and began flowering the following spring. These results indicate that the smooth curd morphotype caused by *BobCAL_T* is partially complemented by *BobCAL_Ga*.

The transformed plants were self-fertilized for two generations, and two transgenic lines for the *BobCAL_Ga* gene were obtained. Southern hybridization confirmed that a single copy of the transgene had been inserted into the cauliflower genome (Fig. 4d). The expression of the *BobCAL* gene in cauliflower was analyzed by examining the transcripts of *BobCAL_Ga* in Bc-1 and Bc-2 plants in shoot apices at the curd induction stage by RT-PCR. Two bands, corresponding to 790- and 920-bp fragments, were resolved in an agarose gel (Fig. 4e). The shorter fragments, whose lengths were consistent with *BobCAL_Ga* cDNAs, exhibited higher expression than *sc-1*, whereas the longer fragments, corresponding to *BobCAL_Tc* and *BobCAL_Td* cDNAs, displayed lower expression levels. These results suggest that exogenous *BobCAL*_*Ga* was overexpressed in *sc-1* plants (Fig. 4f). At the same time, the reduced expression of *BobCAL_Tc* and *BobCAL_Td* in Bc-1 and Bc-2 plants implied that the overexpression of *BobCAL_Ga* decreased the expression levels of *BobCAL_Tc* and *BobCAL_Td*. We conclude that *BobCAL_Ga* conferred a floral development function in smooth curd accessions.

*BobLFY* and *BobAG* were upregulated in Bc-1 and Bc-2 plants (Fig. 4f, g), revealing that *BobCAL_Ga* positively regulates *BobLFY* and *BobAG* in cauliflower. The transition from a smooth curd morphotype to a hairy-like morphotype may be caused by the upregulation of *BobLFY* and *BobAG*.

## Discussion

### Granular and hairy curd morphotypes are relevant to the AS of *BobCAL-G*

In plants of *A. thaliana*, floral meristems are produced from inflorescence meristems after floral induction^28,29^. In cauliflower, inflorescence meristems are temporarily arrested, as they fail to produce floral meristems, with numerous inflorescence meristems then appearing on the curd surface. We found that these events occur in tightly compact curds whose inflorescence meristems are naked and arrested. Unlike smooth and coarse curds, granular curds include numerous floral meristems that are temporarily arrested, while hairy curds retain the state of floral buds on the curd surface. Most likely, floral meristem arrest occurs in granular curds after the cessation of inflorescence meristem arrest, whereas hairy curds experience floral organ arrest after the interruption of inflorescence and floral meristem arrest. The inflorescence meristem arrest of granular and hairy curds is thus of shorter duration than that of smooth and coarse curds.

The mutation of *BobCAL* was previously considered to be strongly associated with the curd phenotype^24^. However, the goodness-of-fit test rejected this model by showing that *BobCAL* alleles were poor predictors of curd phenotypes^26^. The contradiction about the role of *BobCAL* in curd formation apparently arose because the surveyed accessions with different curd morphotypes were mixed together. We discriminated accessions with hairy and granular curds from those with smooth and coarse curds and found that smooth and coarse curd morphotypes are highly associated with the 451T SNP (*BobCAL_T*), whereas hairy and granular curd morphotypes are correlated with 451G (*BobCAL_G*). Although *sc* and *cc* accessions differ in regard to the presence of cauline leaves subtended in curds, they share the same G451T SNP. In theory, *BobCAL_T* alleles encode putative truncated proteins in *sc* and *cc* accessions, whose inflorescence meristems are arrested irrespective of the splice variants of *BobCAL_T*. In contrast, *BobCAL_G* alleles are expected to encode normal proteins in *hc* and *gc* accessions, whose floral meristems and floral organs are arrested in a manner dependent on the splice variants of *BobCAL_G*. Thus, the AS of *BobCAL_G* is crucial for the granular and hairy curd morphotypes of *gc* and *hc* accessions. However, the hairy phenotype of *hc-6* is not linked to *BobCAL_G*. In *hc-6* plants, the ratio of *BobCAL_T* (*a* + *b*) to (*c* + *d*) is >1.5. Most likely, the altered function of the other genes can compensate for the loss of *BobCAL* function in this accession. On the other hand, the smooth and coarse phenotypes of accessions *sc-3*, *sc-14*, and *cc-10* are not linked to *BobCAL_T*. We suggest that the splice variants of *BobCAL_G* are not the sole reason for the occurrence of hairy curds and that the S451T SNP is not the sole element responsible for the occurrence of smooth and coarse curds.

We observed curd surfaces at the time of curd maturity that is ideal for harvesting. At that time, the accessions with compact and smooth curds had not shown any riceyness. However, some compact and smooth curds tended toward certain degrees of riceyness post harvest in spring. “Riceyness” refers to precocious flower bud initiation on the curd surface of cauliflower, which is undesirable for the market^30^. The difference in the degree of riceyness in compact and smooth curds is reflected by the distribution frequency of the flowering time. The plants within the whole population flowered earlier on average in 2016 than in 2017. This implies that riceyness and curd compactness are affected by the interaction between genes and environmental elements, as flowering times are altered under different growing conditions.

### Relative levels of *BobCAL_Ga* and *BobCAL_Gb* transcripts define the fates of floral meristem and floral organ arrest

Among the accessions with *BobCAL_G* alleles in our study, the total expression levels of the four AS variants in plants with granular curds were almost equal to those in hairy curd plants. This observation strongly suggests that *BobCAL_G* genes in *gc* and *hc* accessions show only partial activities. If the *BobCAL_G* genes were fully functional, the inflorescences would not have been arrested and would have developed into normal flowers, as observed in cabbage. If the *BobCAL_G* genes had completely lost their functionality, the inflorescences would have been arrested, as observed in smooth curds. Compared with *sc* and *cc* accessions, which are characterized by inflorescence meristem arrest, *gc* and *hc* accessions are marked by floral meristem arrest and floral organ arrest, respectively, apparently because of the partial function of *BobCAL_G* alleles.

The total expression levels of the four different *BobCAL_G* AS transcripts are roughly equal between *hc* and *gc* plants. In this situation, any change in the expression level of one AS variant should affect the expression levels of the others. In *gc* accessions, both *BobCAL_Ga* and *BobCAL_Gb* are expressed at lower levels than *BobCAL_Gc* and *BobCAL_Gd*, and their levels are sufficient to overcome inflorescence meristem arrest but not floral meristem arrest. In *hc* accessions, *BobCAL_Ga* and *BobCAL_Gb* are expressed at levels higher than *BobCAL_Gc* and *BobCAL_Gd*, and their expression is sufficient to overcome both inflorescence and floral meristem arrest but not floral organ arrest. The relative level of *BobCAL_G* (*a* + *b*) determines the fate of inflorescence meristem arrest, floral meristem arrest and floral organ arrest and, thus, the curd morphotype. Granular and hairy curd morphotypes are caused by floral meristem arrest and floral organ arrest, respectively.

Under normal conditions, the expression levels of *BobCAL_G* (*a* + *b*) relative to *BobCAL_G* (*c* + *d*) are stable in each *hc* and *gc* accession. When *BobCAL_Ga* and *BobCAL_Gb* transcripts are predominantly expressed, floral organ arrest occurs rather than floral meristem arrest. When the expression of *BobCAL_Gc* and *BobCAL_Gd* transcripts is predominant, the opposite is true. We verified this outcome via the genetic transformation of *BobCAL_Ga* in cauliflower. The overexpression of *BobCAL_Ga* decreased the expression levels of *BobCAL_Tc* and *BobCAL_Td* and caused a transition from inflorescence meristem arrest to floral organ arrest. In the field, environmental conditions such as temperature and photoperiod usually affect curd morphotypes and cauliflower yield in *gc* and *hc*-like cultivars, probably by influencing the expression levels of *BobCAL_G* (*a* + *b*) relative to *BobCAL_G* (*c* + *d*).

The question arises of how *gc* and *hc* accessions control the expression levels of *BobCAL_Ga* and *BobCAL_Gb* relative to those of *BobCAL_Gc* and *BobCAL_Gd*. One possibility is that some elements upstream of *BobCAL_Ga* and *BobCAL_Gb* variants differentially regulate one or more AS events. Alternatively, the partial or complete retention of introns 4 and/or 7 in *BobCAL_Gc* and *BobCAL_Gd* may influence their expression in some accessions.

### Curd morphotypes of cauliflower are regulated by the AS of *BobCA*L alleles

In Arabidopsis, an increase in *LFY* expression and the consequent suppression of *TFL1* initiates flowering via the upregulation of *AP1* and *CAL*. *ap1 cal* double mutants have a ‘cauliflower’ appearance^11^, while the corresponding single mutants do not. If cauliflower plants form curds in the same manner, the activity of *AP1* must not be complete, as a single *BobCAL* mutation is insufficient to alter the curd phenotype. Therefore, it is important to clarify whether *BobAP1* is fully activated in different cauliflower accessions.

In cauliflower plants, inflorescence meristem arrest is the primary cause of smooth and coarse curd morphotypes, while floral meristem arrest and floral organ arrest are responsible for granular and hairy curd morphotypes, respectively. *BobCAL*_*T* is nearly fixed in *sc* and *cc* accessions, and the same is true for *BobCAL*_*G* in *gc* and *hc* accessions. Either *BobCAL*_*T* or *BobCAL*_*G* alleles generate the four types of splice variants: *a*, *b*, *c*, and *d*. The predominant expression of *BobCAL*_*Ga* and/or *BobCAL*_*Gb* favors the hairy curd morphotype, whereas the predominant expression of *BobCAL*_*Gc* and/or *BobCAL*_*Gd* results in granular curds. To clarify the genetic relationship between curd morphotypes and the relative expression levels of *BobCAL* variants, their (*a* + *b*)/(*c* + *d*) ratios were compared. We found that the accessions with *BobCAL*_*T* showed smooth and/or coarse curds regardless of the ratio of (*a* + *b*)/(*c* + *d*), whereas the accessions with *BobCAL*_*G* exhibited granular or hairy curds. When the (*a* + *b*)/(*c* + *d*) ratio of *BobCAL*_*G* is >1.5, all accessions show hairy curds, and when the ratio is ≦1.5, all accessions show granular curds. Clearly, the (*a* + *b*)/(*c* + *d*) ratio of *BobCAL*_*G* is relevant to the fate of hairy and granular phenotypes.

**Fig. 5:**
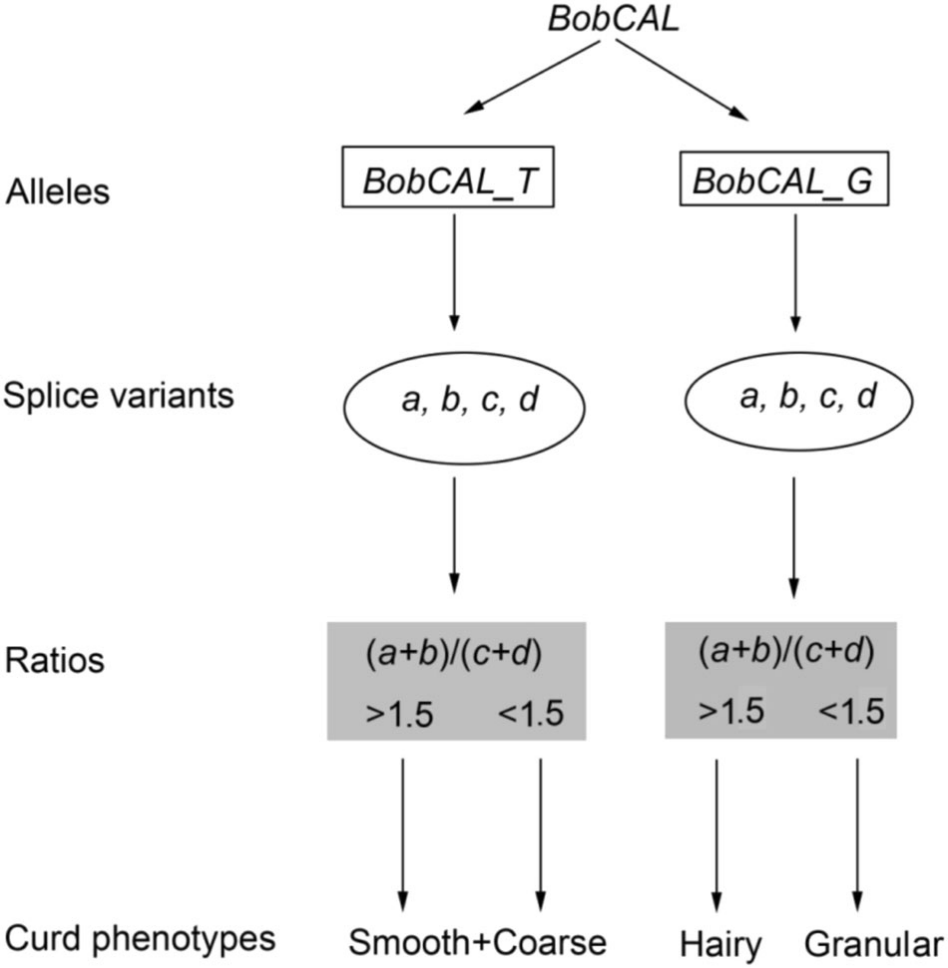
Proposed model of the genetic regulation of cauliflower curd phenotypes by the alternative splicing of the *BobCAL* gene.

On the basis of our genetic and expression analysis results, we propose a model for the genetic regulation of *BobCAL* gene AS to explain the curd phenotypes of cauliflower (Fig. 5). In this model, the *BobCAL* genes present two copies of *BobCAL_T* and *BobCAL-G*, each of which shows four types of AS variants. *BobCAL_T* mainly exists in *sc* and *cc* accessions, while *BobCAL-G* is found in *gc* and *hc* accessions. Smooth and/or coarse curds are associated with *BobCAL*_*T* regardless of the ratio of (*a* + *b*)/(*c* + *d*), whereas granular and hairy curds are associated with *BobCAL*_*G*. In the latter case, higher ratios of (*a* + *b*)/(*c* + *d*) are associated with hairy curds, while lower ratios are associated with granular curds. The (*a* + *b*)/(*c* + *d*) ratios of *BobCAL*_*G* determine the fate of hairy and granular curds.

The plants with the four types of curds show differences in vernalization to initiate flowering. The *sc-2* plants with smooth curds flower latest owing to the delayed transition from inflorescence-to-floral meristems. They require a longer period of vernalization for flowering. The transgenic Bc-1 and Bc-2 plants do not require vernalization and bolt and flower before winter. This difference indicates that the relative abundance of *BobCAL_Ga* variants not only alters curd morphotypes in cauliflower but also affects the timing of the transition from inflorescence-to-floral meristems and therefore affects the fate of vernalization in cauliflower plants.

## Materials and methods

### Plant materials and growth conditions

A total of 78 cauliflower accessions with different curd phenotypes were used in this study. All accessions were self-fertilized for more than five generations to obtain inbred lines. Cauliflower seeds were sown in July, and the resultant seedlings were transplanted into the field one month after germination according to conventional practices at the SIPP Experimental Station, Shanghai, China. The field experiments were conducted in a randomized complete block design with two replications^31^ in replicated field trials for three successive growing seasons (July 2016–2018). More than 20 plants per accession were transferred into greenhouses in the subsequent January for further growth. All accessions were phenotyped for curd-related traits.

Transgenic cauliflower plants were grown simultaneously in the field in July 2017 and transplanted into a greenhouse in the following January. Two lines of T_1_ generation transgenic cauliflower plants and their positive progenies were self-pollinated, and seeds were harvested at maturity. For segregation testing, seedlings from the T_2_ and T_3_ generations were selected on agar-solid Murashige–Skoog (MS) medium with 50 mg/L kanamycin (Km).

### Phenotype characterization

The stage of developmental arrest was scored on a four-point scale with a photographic reference card^32^ and coded into four phenotypes ranging from inflorescence meristem arrest (smooth and coarse curds) to floral meristem arrest (granular curds) and floral organ arrest (hairy curds). The number of days to flowering was counted from transplantation to the opening of the first flower bud in plants. The number of days to curd maturity was counted from transplantation to the harvest maturity of curds. The width of curds was measured to represent curd size.

### Scanning electron microscopy

Shoot apices with developing leaves (5 mm in length) were harvested from the plants of different types at the curd induction stage. For scanning electron microscopy, the tissues were fixed in FAA (2% formaldehyde, 44.5% ethanol, and 6% acetic acid), dehydrated, critical point-dried with CO_2_, and sputter-coated with gold palladium. Specimens from at least three plants for each variant were observed and photographed with a JEOL JSM-6360LV scanning electron microscope.

### Vector construction

A 0.8-kb EcoRI fragment containing *BobCAL_Ga* cDNA was cloned from an RNA sample isolated from the hairy morphotype accession *hc-1* and subcloned into the EcoRI site of a pBluescript KS plasmid. The insertion orientation of *BobCAL_Ga* was verified by cleaving a pre-existing XhoI site in the gene. The *BobCAL_Ga* cDNA was then excised using BamHI and KpnI and inserted into a pJR1 binary vector between the CaMV 35S promoter and the NOS terminator. The resulting plant expression vector, *pJR-BobCAL_Ga*, was subsequently introduced into *Agrobacterium* LBA4404 via the freeze-thaw method^33^.

### Generation of transgenic cauliflower

Seeds of the smooth-morphotype inbred line *sc-1* were surface sterilized and then germinated on agar-solid MS medium. Hypocotyl segments of 8–10-day-old seedlings were infected with LBA4404 containing *pJR-BobCAL_Ga* and cocultured for 3 days. The infected segments were then induced to produce Km-resistant shoots on MS medium containing 3 mg/L 6-BA, 0.5 mg/L IAA, 500 mg/L carbenicillin and 50 mg/L Km. The positive shoots were induced to root on MS medium containing 0.5 mg/L IAA and then transplanted into the greenhouse.

Three Km-resistant lines were identified as transgenic by PCR and Southern hybridization. The primers used for PCR amplification were npt-p1 (5′-GAGGCTATTCGGCTATGACTG-3′) and npt-p2 (5′-ATCGGCAGCGGCGATACCGTA-3′), which were specific for the NPT-II gene conferring Km resistance in the vector pJR1. For Southern hybridization, 10 µg of genomic DNA extracted from untransformed and Km-resistant cauliflower leaves was digested with EcoRI and BamHI and then subjected to 1.0% agarose gel electrophoresis. A PCR-amplified fragment of the 35S promoter labeled with ^32^P was applied as a probe to detect plant genomic DNA following the procedure of Sambrook et al.^4^.

### RNA and DNA extraction

For the cDNA cloning and RNA isoform analysis of the *BobCAL*, *BobLFY*, *BobAP1*, and *BobAG* alleles, shoot tips with developing leaves were freshly harvested at the curd induction stage, and RNA samples were isolated from shoot tip tissues. For RT-PCR and real-time PCR, three plants of each accession at the same stage as mentioned above were randomly selected for the sampling of shoot tips with developing leaves, from which RNA samples were isolated. Ten accessions were randomly selected to perform real-time PCR for the discrimination of expression levels between the four groups of accessions.

Total RNA was extracted from shoot tips at the curd induction stage via the guanidinium isothiocyanate method^34^. Total genomic DNA was extracted from cauliflower using a modified CTAB method^35^.

### RT-PCR

Two micrograms of total RNA was reverse transcribed and amplified using an mRNA Selective Reverse Transcription 2.1 PCR kit (Takara, Dalian, China) according to the manual provided by the manufacturer^34^.

The primers used for the RT-PCR analysis of *UBQ*, *BobTFL1*, *BobLFY*, *BobAP1*, *BobCAL*, and *BobAG* are listed in Supplementary Table 1. The annealing temperatures used for PCR amplification were as follows: 54 °C for *BobTFL1*, *BobLFY*, and *BobAP1* and 56 °C for *BobCAL*, *BobAG*, and *UBQ*. Specific primer pairs (Supplementary Table 3) were designed to perform real-time PCR in a MyiQ2 Two-color Real-time PCR Detection System (Bio-Rad, Richmond, CA, USA). For the expression analysis of each splice variant, the RT-PCR/real-time PCR experiments were performed more than three times, and the methods reproducibly determined the expression levels and experimentally validated the expression levels. At least three biological replicates of the quantitative PCR assays were performed for each gene. The relative transcript level of each gene was normalized to that of *BobUBQ* cDNA for quantification. *t*-tests were performed to analyze the significance of differences.

### Statistics analysis

For real-time PCR, three biological replicates of the quantitative PCR assays were performed for each accession. The relative transcript level of each gene was normalized to the *BobUBQ* cDNA level for quantification^34^. Error bars indicate the standard error. For boxplot analysis, all data are presented as the mean ± SE (*n* ≧ 10 accessions). Clusters with the same letters are not significantly differentiated from each other at the 0.01 level according to the *t*-test^36^.

### Identification of *BobCAL* gene AS variants

After the reverse transcription of 2 µg of RNA using oligo-dT primers with reverse transcriptase (Takara), 5 μL of the resulting products were subjected to PCR amplification with the BobCAL-F/BobCAL-R primers as described above. The electrophoresis of the products in a 1% agarose gel yielded two specific bands. Both bands were recovered and cloned into a pMD18-T vector (Takara) for sequencing.

### Sequence analysis

All cDNA sequences (GenBank: AY514052–AY514055) were analyzed with DNAStar Lasergene sequence analysis software and then aligned in CLUSTAL X 1.8^36^.

### Association analysis

Box plots were drawn according to the method of Tukey^37^. The median and quartile values of gene expression data of the cauliflower accessions were calculated using R statistical software. Significant differences between subpopulations were detected by the Kruskal–Wallis test^38^.

## Supplementary information

Supplemental Table 1

Supplementary Table 2

Supplemental Table 3

Supplementary Figure 1

## References

[1] Smyth DR (1995). Flower development: origin of the cauliflower. Curr. Biol..

[2] Kieffer M, Fuller M, Jellings A (1996). Mathematical model of cauliflower curd architecture based on biometrical analysis. Acta Hortic..

[3] Sadik S (1962). Morphology of the curd of cauliflower. Am. J. Bot..

[4] De Candolle, A. *Prodromus* (Treuttel and Wurtz, 1824).

[5] Masters MT (1869). On the structure of the flower in the genus *Napoleona*, &c. Botanical J. Linn. Soc..

[6] Sadik S, Ozbun J (1968). The association of carbohydrate changes in the shoot tip of cauliflower with flowering. Plant Physiol..

[7] Anthony RG, James PE, Jordan BR (1996). Cauliflower (*Brassica oleracea* var. *botrytis* L.) curd development: the expression of meristem identity genes. J. Exp. Bot..

[8] Carr SM, Irish VF (1997). Floral homeotic gene expression defines developmental arrest stages in *Brassica oleracea* L. vars. *botrytis anditalica*. Planta.

[9] Bowman JL (1993). Control of flower development in *Arabidopsis thaliana* by APETALA1 and interacting genes. Development.

[10] Kempin SA, Savidge B, Yanofsky MF (1995). Molecular basis of the cauliflower phenotype in *Arabidopsis*. Science.

[11] Ferrandiz C, Gu Q, Martienssen R, Yanofsky MF (2000). Redundant regulation of meristem identity and plant architecture by FRUITFULL, APETALA1 and CAULIFLOWER. Development.

[12] Alvarez-Buylla ER, Garcia-Ponce B, Garay-Arroyo A (2006). Unique and redundant functional domains of APETALA1 and CAULIFLOWER, two recently duplicated *Arabidopsis thaliana* floral MADS-box genes. J. Exp. Bot..

[13] Pelaz S (2000). B and C floral organ identity functions require SEPALLATA MADS-box genes. Nature.

[14] Pelaz S (2001). APETALA1 and SEPALLATA3 interact to promote flower development. Plant J. Cell Mol. Biol..

[15] Samach A (2000). Distinct roles of CONSTANS target genes in reproductive development of *Arabidopsis*. Science.

[16] Hartmann U (2000). Molecular cloning of SVP: a negative regulator of the floral transition in *Arabidopsis*. Plant J. Cell Mol. Biol..

[17] Krizek BA, Meyerowitz EM (1996). Mapping the protein regions responsible for the functional specificities of the *Arabidopsis* MADS domain organ-identity proteins. Proc. Natl Acad. Sci. USA.

[18] Riechmann JL, Krizek BA, Meyerowitz EM (1996). Dimerization specificity of *Arabidopsis* MADS domain homeotic proteins APETALA1, APETALA3, PISTILLATA, and AGAMOUS. Proc. Natl Acad. Sci. USA.

[19] Fan HY, Hu Y, Tudor M, Ma H (1997). Specific interactions between the K domains of AG and AGLs, members of the MADS domain family of DNA binding proteins. Plant J..

[20] Mizukami Y (1996). Functional domains of the floral regulator AGAMOUS: characterization of the DNA binding domain and analysis of dominant negative mutations. Plant Cell.

[21] Moon Y-H (1999). Determination of the motif responsible for interaction between the rice APETALA1/AGAMOUS-LIKE9 family proteins using a yeast two-hybrid system. Plant Physiol..

[22] Yang Y, Jack T (2004). Defining subdomains of the K domain important for protein-protein interactions of plant MADS proteins. Plant Mol. Biol..

[23] Huang H (1995). The *Arabidopsis* MADS-box gene AGL3 is widely expressed and encodes a sequence-specific DNA-binding protein. Plant Mol. Biol..

[24] Smith LB, King GJ (2000). The distribution of BoCAL-a alleles in *Brassica oleracea* is consistent with a genetic model for curd development and domestication of the cauliflower. Mol. Breed..

[25] Purugganan MD, Boyles AL, Suddith JI (2000). Variation and selection at the CAULIFLOWER floral homeotic gene accompanying the evolution of domesticated *Brassica oleracea*. Genetics.

[26] Labate JA, Robertson LD, Baldo AM, Björkman T (2006). Inflorescence identity gene alleles are poor predictors of inflorescence type in broccoli and cauliflower. J. Am. Soc. Hortic. Sci..

[27] Ratcliffe OJ, Bradley DJ, Coen ES (1999). Separation of shoot and floral identity in *Arabidopsis*. Development.

[28] Kieffer M, Davies B (2001). Developmental programmes in floral organ formation. Semin. Cell Dev. Biol..

[29] Pidkowich MS, Klenz JE, Haughn GW (1999). The making of a flower: control of floral meristem identity in *Arabidopsis*. Trends Plant Sci..

[30] Zhao Z (2020). Identification of candidate genes involved in curd riceyness in cauliflower. Int. J. Mol. Sci..

[31] Yousef EAA, Lampei C, Schmid KJ (2015). Evaluation of cauliflower genebank accessions under organic and conventional cultivation in Southern Germany. Euphytica.

[32] Labate JA, Robertson LD, Baldo AM (2006). Inflorescence identity gene alleles are poor predictors of inflorescence type in broccoli and cauliflower. J. Am. Soc. Hortic. Sci..

[33] Chen H, Nelson R, Sherwood J (1994). Enhanced recovery of transformants of *Agrobacterium tumefaciens* after freeze-thaw transformation and drug selection. Biotechniques.

[34] Sambrook J, Fritsch E, Maniatis T (1989). Molecular Cloning: A Laboratory Manual.

[35] Porebski S, Bailey LG, Baum BR (1997). Modification of a CTAB DNA extraction protocol for plants containing high polysaccharide and polyphenol components. Plant Mol. Biol. Rep..

[36] Thompson JD (1997). The CLUSTAL_X windows interface: flexible strategies for multiple sequence alignment aided by quality analysis tools. Nucleic Acids Res..

[37] Tukey, J. W. *Exploratory Data Analysis* (Addison-Wesley, Reading, 1977).

[38] Kruskal WH, Wallis WA (1952). Use of ranks in one-criterion variance analysis. J. Am. Stat. Assoc..

