# Supplementary material for: Alternatively spliced BobCAL transcripts alter curd morphotypes in a collection of Chinese cauliflower accessions
Source: Hortic Res. 2020 Oct 1;7:160. doi: 10.1038/s41438-020-00378-x (PMC7527968; doi:10.1038/s41438-020-00378-x)
Supplement: Supplementary file 1 — Supplemental Table 1 [file 41438_2020_378_MOESM1_ESM.docx]

Supplemental Table 3. Primer pairs used in this study

| Primer | Sequence | Usage |
| --- | --- | --- |
| BobCAL-E4_F  BobCAL-E4_R | 5'-GCATTATCTGGGAGAAGATT  5'-TCTGGAGCGAATATGTTTAAG | RT-PCR of *BobCAL* exon 4 |
| BobCALa_F  BobCALa_R | 5'-GCTCCAGAAAAAATCAACT-3'  5'-ACATGCCACCCATATTTAG-3' | Real-time PCR of *BobCAL_Ga* |
| BobCALc_F  BobCALc_R | 5'-GCTCCAGAAAAGTGTGTAA-3'  5'-ATTAGTTGATTCTGCATAC-3' | Real-time PCR of *BobCAL_Gc* |
| BobCALab_F  BobCALab_R | 5'-GCTCCAGAAAAAATCAACT-3'  5'- TATTCTCCTTGGTACATGC-3' | RT-PCR of *BobCAL_Ga* and *b* |
| BobCAL_F  BobCAL_R | 5'-TCTACGAGAAATGGGAAGG-3'  5'-GTCGATATATGGCGAGTCC-3' | Cloning and RT-PCR of *BobCAL* coding sequence |
| BobTFL1_F  BobTFL1_R | 5'-AATGGAGAATATGGGAACTAGAG-3'  5'-GTAACTAACGTCTGCGAGATGC-3' | Real-time PCR of *BobTFL1* |
| BobAP1_F  BobAP1_R | 5'-GCACATCCGCTCTAGAAAAAACCAAC-3'  5'-CTTCTTGATACAGCCCTCCCATGT-3' | Real-time PCR of *BobAP1-a* |
| BobAP2_F  BobAP2-R | 5'-GCTGCAGCATCATCAGGATTCT-3'  5'-AGTTAATTTAGTGACCAAGACC-3' | Real-time PCR of *BobAP2* |
| BobAP3_F  BobAP3-R | 5'-AGGCTGGTTCTACTACAACCA-3'  5'-TCCACAATAAGTCAAAGCAGC-3' | Real-time PCR of *BobAP3* |
| BobAG_F  BobAG_R | 5'-TTGATGGGTGAGACGATTGGGT-3'  5'-ACGGTTGAGGTTGCGTTTGAGG-3' | Real-time PCR of *BobAP1-a* |
| BobLFY_F  BobLFY_R | 5'-GGGTTTGTTCGGTCCTTACG-3'  5'-ACGGGTGCTCCCTTTGTCTC-3' | Real-time PCR of *BobLFY* |
| BobUBQ_F  BobUBQ_R | 5'-GGTGCTAAGAAGAGGAAGAAT-3'  5'-CTCCTTCTTTCTGGTAAACGT-3' | Real-time PCR of *BobUBQ* |
