# Supplementary material for: Alternatively spliced BobCAL transcripts alter curd morphotypes in a collection of Chinese cauliflower accessions
Source: Hortic Res. 2020 Oct 1;7:160. doi: 10.1038/s41438-020-00378-x (PMC7527968; doi:10.1038/s41438-020-00378-x)
Supplement: Supplementary file 4 — Supplementary Figure 1 [file 41438_2020_378_MOESM4_ESM.docx]

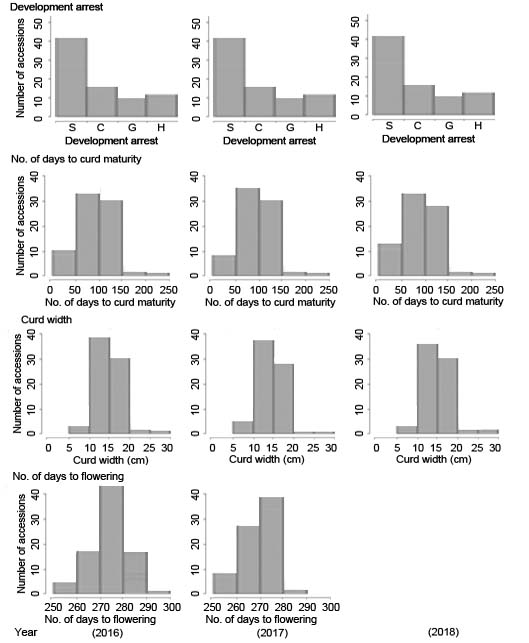


Supplementary Figure 1. Frequency distribution of curd-related traits in a cauliflower collection of 78 accessions.
